# Casein Kinase 1 Delta Regulates Cell Proliferation, Response to Chemotherapy and Migration in Human Ovarian Cancer Cells

**DOI:** 10.3389/fonc.2019.01211

**Published:** 2019-11-12

**Authors:** Elena Laura Mazzoldi, Anna Pastò, Elisa Ceppelli, Giorgia Pilotto, Vito Barbieri, Alberto Amadori, Simona Pavan

**Affiliations:** ^1^Immunology and Diagnostic Molecular Oncology Unit, Veneto Institute of Oncology, IOV-IRCCS, Padua, Italy; ^2^Department of Surgery, Oncology and Gastroenterology, University of Padua, Padua, Italy

**Keywords:** epithelial ovarian cancer, casein kinase 1 delta, cell proliferation, carboplatin, cell migration

## Abstract

Casein kinase 1 delta (CK1δ) has a tumor-promoting role in different cancers and it is genetically amplified in a portion of human epithelial ovarian cancer (EOC). CK1δ is involved in pleiotropic cellular functions such as cell proliferation, DNA damage, and migration. We specifically knocked down CK1δ by short hairpin RNA (shRNA) in human ovarian cancer cells and we performed proliferation, chemosensitivity, as well as *in vitro* and *in vivo* migration assays. CK1δ knocked-down cells displayed reduced proliferation capability both *in vitro* and *in vivo*. Nonetheless, these cells were sensitized to the first line chemotherapeutic agent carboplatin (CPT), and this observation could be associated to reduced expression levels of p21(Cip1/Waf1), involved in DNA damage response, and the anti-apoptotic X-linked inhibitor of apoptosis protein (XIAP). Moreover, CK1δ knocked-down cells were affected in their migratory and lung homing capability, even if in opposite ways, i.e., IGROV1, SKOV3 and MES-OV lost, while OVCAR3 gained motility potential. The results suggest CK1δ as a potential exploitable target for pharmacological EOC treatment, but they also advise further investigation of its role in cell migration.

## Introduction

Epithelial ovarian cancer (EOC) is the first cause of death if considering only the gynecological malignancies, and the fifth leading cause of cancer-related death among women (1). This mortality is mainly due to the paucity of symptoms and to the lack of an effective screening program; as a consequence, EOC is usually diagnosed at advanced stages (2). EOC is often considered chemosensitive, with a response rate higher than 80% after first-line treatments. However, about 70% of patients relapse within 18 months (3). Recently, PARP [poly (ADP-ribose) polymerase] inhibitors such as Olaparib and Niraparib have been approved for treatment of BRCA-deficient EOC patients who have completely or partially responded to chemotherapy treatments (4). Nevertheless, the treatment of remaining EOC patients is still challenging and the search for possible molecular targets could help in the identification of promising therapeutic options.

Casein kinases are a group of evolutionarily conserved serine/threonine kinases ubiquitously expressed in eukaryotes. This group includes two families: casein kinase 1 (CK1) and casein kinase 2 (CK2) (5). Six CK1 genes, i.e., CK1 α, γ1, γ2, γ3, δ, and ε have been identified in humans. All the isoforms display a high homology in their catalytic domain. For instance, the highly related CK1δ and ε are 98% identical in their kinase domain, but they differ in the regulatory N-terminal and C-terminal domains. CK1 are monomeric, constitutively active, co-factor independent kinases (6). CK1 recognize a consensus sequence S/T(P)-(X)_1−2_-S/T for phosphorylation. The substrates targeted by the different CK1 are involved in a plethora of cellular functions, including cell cycle progression, chromosome segregation, apoptosis, DNA repair, circadian rhythm, ribosome biogenesis, vesicle trafficking, p53, Wnt, Shh, and Hippo pathways (7–10).

CK1 activity leads to anti-apoptotic effects, in many different ways. Indeed, CK1α mediates the resistance to TRAIL (tumor necrosis factor related apoptosis inducing ligand) by phosphorylating members of the death-inducing signaling complex (DISC). CK1α, δ, and ε phosphorylate the pro-apoptotic protein BID, thus reducing its caspase 8-mediated cleavage and activation (8).

CK1δ has been shown to be implicated in mitotic spindle dynamics and cell cycle progression (6). Indeed, CK1δ expression increases as cell cycle progresses from G1/S to G2/M phase, in order to efficiently phosphorylate and lead to proteasomal degradation the tyrosine kinase Wee1, which is a negative regulator of CDK1-cyclin B1, the checkpoint that controls the mitotic entry (11). Moreover, CK1δ regulates the total level and phosphorylation of the checkpoint kinase Chk1, which is induced in response to DNA damage. As a consequence, CK1δ silencing disrupts the Chk1-dependent G2/M checkpoint (7, 12).

Alteration in the expression of CK1 has been observed in cancer (13, 14). However, CK1 mutations seem to be rare in The Cancer Genome Atlas (TCGA) set of cancers (8). Additionally, the detected mutations do not accumulate in hotspots (8). Nonetheless, copy number variations are more frequently found. For example, in ovarian cancer, CK1δ gene (*CSNK1D*) is amplified in about 4% of the cases reported and is much more rarely deleted, while point mutations are not recorded (15, 16). CK1δ is overexpressed in cells of hyperplastic B cell follicles and B cell lymphomas in p53-deficient mice (17), as well as in human choriocarcinoma (18), and in pancreas ductal adenocarcinoma (6, 8, 19). In breast cancer, CK1δ is strongly expressed in low grade carcinomas and is reduced in less differentiated cancers (6). Moreover, forced expression of a dominant-negative mutant CK1δ impairs SV40-induced transformation of mammary cells (20).

The development of CK1δ isoform-specific inhibitors to be used in the clinical practice has been undertaken. To date, different ATP-competitive inhibitors have been developed, but they have shown no specificity for the CK1δ or ε isoform with further limitations such as off-targets effects, limited anti-proliferative activity in cell-based assays, poor solubility and pharmacokinetic properties (20). Therefore, in order to dissect if the possible effects can be ascribed to CK1δ or CK1ε inhibition, it is still necessary to study the specific role of the two kinase isoforms by genetic ablation by means of selective siRNA or shRNA.

Herein, we have investigated the role of CK1δ in ovarian cancer by specific short hairpin RNA approach, and addressed its role in proliferation, chemosensitivity and cell motility. Notably, we have shown that CK1δ knockdown affects ovarian cancer cell growth, and it sensitizes cells to carboplatin treatment. Moreover, CK1δ ablation impacts negatively on cell migration, even if with one exception.

## Materials and Methods

### Cell Lines and *in vitro* Culture

Human ovarian cancer cell lines OVCAR3 and SKOV3, and embryonic kidney HEK293T cells were purchased from ATCC (Manassas, VA). Human ovarian cancer cell line IGROV1 was kindly provided by Prof. Silvana Canevari (Istituto Nazionale dei Tumori, Milan, Italy). Human ovarian cancer cell line MES-OV, its carboplatin-resistant derivative MES-OV CBP, and carboplatin-resistant OVCAR3 CBP cells were kindly provided by Dr. Anamaria Brozović (Ruder Bošković Institute, Zagreb, Croatia) (21). OVCAR3, OVCAR3 CBP, IGROV1, and SKOV3 cells were cultured in RPMI-1640 medium (Euroclone, Milan, Italy), supplemented with 10% fetal bovine serum (FBS; GIBCO, Thermo Fisher Scientific, Waltham, MA), 100 U/mL Penicillin/Streptomycin (Lonza, Basel, Switzerland), 1 mM sodium pyruvate (Lonza), and 2 mM Ultraglutamine (Lonza). Cells were cultured at 37°C, 5% CO_2_, and harvested at confluence using trypsin-EDTA (GIBCO). HEK293T, MES-OV, and MES-OV CBP were cultured in DMEM medium (Euroclone) supplemented as described above.

### Bioinformatic Analysis of CK1δ Expression in the Ovarian Tissue

The expression data of CK1δ gene (*CSNK1D*) and protein were obtained from Oncomine™^1^ (22) and The Human Protein Atlas^2^ (23) platforms, respectively. Fold changes of *CSNK1D* mRNA expression in EOC over normal ovary tissues and the respective *p*-values were retrieved from the TCGA, Bonome, Lu, Hendrix, Adib, and Toshihara ovarian datasets within Oncomine™ database. Immunohistochemistry images of CK1δ in normal ovarian tissue^3^ and in ovarian cancer^4^ were downloaded from the Human Protein Atlas, as well as the description of the protein expression levels.

### Lentiviral Vector Production and Cell Transduction

For *CSNK1D* gene knockdown, MISSION® TRC shRNA bacterial glycerol stocks transformed with plasmids encoding short hairpin RNA (shRNA) specifically targeting human *CSNK1D* (sh599, sh1552) or a scramble control sequence (shCTRL) were purchased from Sigma-Aldrich (St. Louis, MO).

To perform *in vivo* imaging, cells were transduced with the firefly luciferase (Fluc) gene. The plasmid (pHR'EF-Fluc-WSIN) was kindly provided by Dr. Takeya Sato (University of Toronto, Canada).

Lentiviral vector stocks were generated by a transient three-plasmid vector packaging system. Briefly, HEK293T cells were co-transfected with VSV-G construct (pHCMV-G, kindly provided by Prof. Volker Erfle, Institut für Molekulare Virologie, Neuherberg, Germany), pCMVR8.74 (Addgene plasmid #22036, gift from Didier Trono, École Polytechnique Fédérale de Lausanne, Lausanne, Switzerland), and the plasmid of interest. Lentiviral particles were obtained by ultra-centrifugation of cell supernatants (24,000 rpm for 2 h).

For *CSNK1D* knockdown, concentrated virus-containing supernatant was incubated with EOC cell lines, previously seeded into six-well plates at 1.5 × 10^5^ cells/well. After overnight incubation, the supernatant was replaced with fresh complete medium. After 48 h, cells were puromycin-selected (1 μg/mL in OVCAR3, OVCAR3 CBP, MES-OV, and MES-OV CBP cells, 2 μg/mL in SKOV3 cells, and 4 μg/mL in IGROV1 cells, Sigma Aldrich).

For Fluc expression, shCTRL, sh599, and sh1552 OVCAR3 and IGROV1 cells were transduced as described above. To determine bioluminescence intensity, 5 × 10^5^ cells were seeded in black 96-well microplates (Perkin Elmer, Waltham, MA), incubated with D-luciferin (150 ng/mL, Perkin Elmer), or PBS alone as negative control, and subjected to bioluminescence analysis with IVIS Imaging System (Xenogen Corporation, Alameda, CA).

### Patient-Derived Xenograft Generation and *in vivo* Experiments

Non-Obese Diabetic/Severe combined immunodeficiency (NOD/SCID) and NOD/SCID gamma (NSG) mice were obtained from internal breeding.

Patient-derived xenografts (PDX) were generated by injecting NOD/SCID mice intraperitoneally (i.p.) with 10^6^ tumor cells derived from ascitic effusions of EOC-bearing patients (PDOVCA), collected after obtaining written informed consent. Briefly, patients' cancer cells were obtained by centrifugation of the ascitic fluid and subsequent red blood cell lysis, if needed (24). Cells were injected into NOD/SCID mice and ascitic fluid from mice was collected after its accumulation and processed in the same way as patients' clinical samples.

For *in vivo* tumor growth assay, 1 × 10^6^ shCTRL, sh599, and sh1552 OVCAR3 and IGROV1 cells were injected subcutaneously (s.c.) in 200 μl of Matrigel® (Corning, New York, NY) in the dorso-lateral flank of NSG mice, and the growth rate was monitored by caliper measurements. Mice were sacrificed when the tumors of the shCTRL group reached 600–900 mm^3^ volume. For protein extraction, tumors were snap-frozen in liquid nitrogen and homogenized with a T18 basic Ultra-Turrax® disperser (Ika, Staufen im Breisgau, Germany) in RIPA buffer.

For lung colonization assay, 1 × 10^6^ shCTRL, sh599, and sh1552 Fluc-OVCAR3 and IGROV1 cells were injected into the tail vein of NOD/SCID mice. At 2 and 24 h after cell injection, mice received 200 μL of D-luciferin (15 mg/mL) i.p. for 8 min. Then, mice were sacrificed and lungs harvested and subjected to bioluminescence analysis with IVIS Imaging System, as previously described (25).

### RNA Extraction, Reverse Transcription, and Quantitative RT-PCR

Total RNA was extracted following the TRIzol method (Ambion, Thermo Fisher Scientific) as per manufacturer's instruction, as previously described (26). cDNA was retro-transcribed from 1 μg of total RNA using the High capacity RNA-to-cDNA kit (Applied Biosystems, Thermo Fisher Scientific), then it was mixed with Platinum™ SYBR™ Green qPCR SuperMix-UDG (Invitrogen, Thermo Fisher Scientific) and the gene-specific primers; samples were run in duplicate. The PCR reaction was performed on ABI PRISM® 7900HT Sequence Detection System (Applied Biosystems, Thermo Fisher Scientific). ΔΔCt values were utilized to calculate the fold change = 2^−ΔΔ*Ct*^. Data were expressed as the difference in gene expression (normalized to the housekeeping β_2_-microglobulin gene) relative to the shCTRL sample. Primer sequences are: *CSNK1D* (NM_001893) Forward 5′- AGTGTTGTGTAAAGGCTACCC-3′, Reverse 5′-CGAGTAGTCAGGCTTGTCGT-3′; β_2_-microglobulin (NM_004048) Forward 5′-TCTCTCTTTCTGGCCTGGAG-3′; Reverse 5′-TCTCTGCTGGATGACGTGAG-3′.

### Western Blotting (WB)

Cells were lysed with RIPA buffer supplemented with protease (SIGMAFAST™, Sigma-Aldrich) and phosphatase inhibitors (PhosSTOP™, Roche, Basel, Switzerland). Protein concentration was determined by using the bicinchoninic acid (BCA) assay (Quantum Micro Protein, Euroclone). Equal protein amounts were loaded on NuPAGE™ 4–12% Bis-Tris protein precast polyacrylamide gels (Invitrogen, Thermo Fisher Scientific) in denaturing and reducing conditions. Proteins were then transferred onto nitrocellulose membranes (Perkin Elmer). Membranes were saturated with 5% non-fat milk in TBS-Tween 20 buffer, and hybridized with primary antibodies overnight at 4°C. The following primary antibodies were used: CK1δ (1:5,000, Abcam, Cambridge, UK), p21 Waf1/Cip1 (1:1,000, Cell Signaling Technology, Boston, MD), XIAP (1:1,000, Cell Signaling Technology), α-tubulin (1:4,000, Sigma Aldrich), β-actin (1:1,000, Abcam). Primary antibodies were diluted in 5% BSA in TBS-Tween 20 buffer. Secondary horseradish peroxidase (HRP)-conjugated anti-rabbit or anti-mouse antibodies (Perkin Elmer), diluted 1:5,000 in 5% milk in TBS-Tween 20 buffer, were added for 1 h at room temperature. Finally, the chemiluminescence signal was detected with Western Lightning® Plus-ECL (Perkin Elmer) on a ChemiDoc™ XRS Imaging System (Bio-Rad, Hercules, CA), and band densitometry was analyzed by Quantity One® software (Bio-Rad). Signal intensity was normalized either to α-tubulin or β-actin housekeeping proteins.

### Proliferation Assay

ShCTRL, sh599, and sh1552 OVCAR3 and IGROV1 cells were plated in triplicate in four 24-well plates at 5,000 cells/well. Cells were fixed with 4% PFA after overnight culture (day 1) and after 2, 3, and 4 days, and crystal violet-stained (Sigma Aldrich), as reported elsewhere (27). Crystal violet was solubilized in 1% sodium dodecyl sulfate (SDS), and the absorbance was measured at 595 nm using the plate reader Victor™ X4 (Perkin Elmer). The absorbance values were normalized to the corresponding day 1.

### Cell Cycle Analysis

ShCTRL, sh599, and sh1552 OVCAR3 and IGROV1 cells were labeled with 10 μM 5-ethynyl-2′-deoxyuridine (EdU; Baseclick, Neuried, Germany) for 20 min. OVCAR3 and IGROV1 cells were harvested 8 and 7 h later, respectively, and stained following manufacturer's protocol. Cells were subsequently stained with a solution of propidium iodide (100 μg/mL, Sigma-Aldrich) and RNase A (0.8 μg/mL, Qiagen, Hilden, Germany) in 1X saponin-based permeabilization and wash reagent (provided by the kit) at 37 °C for 2 h.

Flow cytometry analysis was performed using a FACS LSRII (BD Bioscience, Franklin Lakes, NJ); data were collected using a low flow rate and elaborated with FlowJo software (TreeStar, Ashland, OR).

### Apoptosis Assay

ShCTRL, sh599, and sh1552 OVCAR3, IGROV1, OVCAR3 CBP, and MES-OV CBP cells treated for 72 h with different doses of carboplatin (5, 10, and 20 μg/mL for OVCAR3 cells, 5, 10, and 15 μg/mL for IGROV1 cells, 50, 75, 100 μg/mL for OVCAR3 CBP cells, 75 and 100 μg/mL for MES-OV CBP cells) were incubated for 15 min at room temperature with Annexin-V-FLUOS (1:50, Roche). Flow cytometry analysis was performed using a FACS LSRII; data were elaborated with FlowJo software.

### *In vitro* Migration Assays

ShCTRL, sh599, and sh1552 OVCAR3, IGROV1, SKOV3 and MES-OV cell migratory capacity was determined *in vitro* by both wound healing and transwell migration assays, as previously described (28).

For wound healing assay, confluent cells were scratched with a p200 pipet tip. Wells were washed to remove detached cells, and medium was replaced with serum-free RPMI-1640 or DMEM (for MES-OV cells). At time 0 and after 24 and 48 h, pictures of the wounded area were taken with Leica DM IL LED microscope (Wetzlar, Germany). The distance between scratch edges was quantified using ImageJ software.

For transwell migration assay, 5 × 10^4^ cells resuspended in 200 μL of RPMI-1640 supplemented with 0.2% FBS were seeded into 8 μm pore cell culture insert (migration chambers, Falcon, Corning) in 24-well plates. Wells were filled with 800 μL of RPMI-1640 medium containing 20% FBS, and cells were incubated at 37°C. After 18 h, cells that had not crossed the membrane were removed with a cotton swab, and inserts were fixed with 4% PFA. Cells on the bottom of the membrane were stained with crystal violet. Images of five fields per insert were taken with a Leica DM IL LED microscope and the area covered by migrated cells was quantified using ImageJ software.

### Statistical Analysis

Data from replicate experiments were shown as mean values ± Standard Deviation (S.D.) or Standard Error of the Mean (S.E.M.), as indicated. Comparisons between groups were done by the two-tail Student's *t*-test and Mann-Whitney test, as appropriate. The association between CK1δ staining score, reported in The Human Protein Atlas, and the status of either normal ovary or cancer tissue was analyzed using χ^2^-test. Statistical analyses were performed by using the Sigmaplot software (Systat Software, San Jose, CA).

### Ethics Approval

Human samples of ascitic effusions from EOC-bearing patients were obtained after receiving written informed consent. The study was approved by IOV Institutional Review Board and Ethics Committee, and was performed in accordance with the declaration of Helsinki.

The animal studies presented in this work were approved by the Italian Ministry of Health (authorization n° 250/2017-PR). Procedures involving animals and their care were performed according to institutional guidelines that comply with national and international laws and policies (EEC Council Directive 86/609, OJ L358, 12 December 1987).

## Results

### CK1δ knockdown Causes a Growth Braking in Human Ovarian Cancer Cells

**Figure 1:**
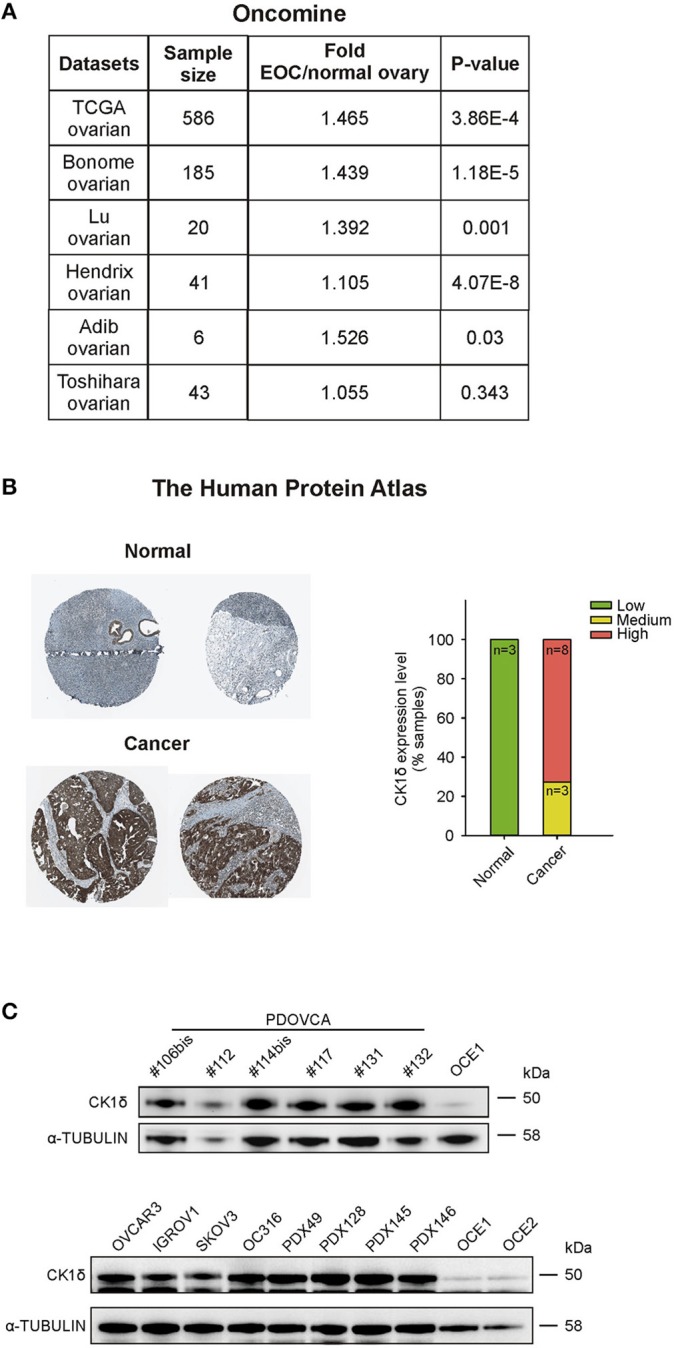
CK1δ expression in human ovarian cancer cells. **(A)** *CSNK1D* gene expression levels in normal ovary and EOC tissues were retrieved from TCGA, Bonome, Lu, Hendrix, Adib, and Toshihara ovarian datasets on the Oncomine™ platform. Sample size, fold changes, and *p*-values are shown in the table. **(B)** Immunohistochemical analyses of CK1δ in normal ovaries and in EOC specimens were obtained from the Human Protein Atlas. On the left, representative pictures downloaded from the website are shown (for further details, see *Data Availability* section). On the right, the graph displays the protein expression level description, as presented in the website (normal tissue: *n* = 3; cancer tissue: *n* = 11 of which three with medium and 8 with high expression). **(C)** Western Blot (WB) analysis of CK1δ in six PDOVCA (106bis, 112, 114bis, 117, 131, 132), four EOC cell lines (OVCAR3, IGROV1, SKOV3, and OC316), four PDX (49, 128, 145 and 146), and two immortalized ovarian epithelial cell lines (OCE1 and OCE2).

CK1δ has been demonstrated to have a pro-tumorigenic role in a variety of cancers, including B cell lymphoma (17), choriocarcinoma (18), pancreas ductal adenocarcinoma (19), colorectal cancer (29) and breast cancer (30). In the latter, CK1δ inhibitors efficiently slowed *in vivo* tumor growth (30). Retrieving expression data from Oncomine™^5^ and The Human Protein Atlas platforms^6^, CK1δ showed a trend toward higher expression levels in ovarian cancer tissue compared to normal ovary at mRNA level (Figure 1A). At the protein level (Figure 1B), the analysis of the CK1δ expression score (low, medium, high) from the Human Protein Atlas showed a statistically significant difference between normal and cancer tissue (*p* < 0.001, χ^2^ test), i.e., a higher expression in the latter.

Thus, we confirmed these observations checking the expression of CK1δ protein in a panel of human primary samples of ovarian cancer (PDOVCA), ovarian cancer cell lines, PDX samples and immortalized ovarian epithelial cell lines (31). As shown in Figure 1C, all the PDOVCA, cell lines and the PDX tested resulted strongly positive for CK1δ compared to the non-tumoral counterpart.

In order to verify whether CK1δ perturbation could have any effect on some typical features of cancer cells, such as proliferation, resistance to apoptosis, migratory ability, and *in vivo* tumor growth, we decided to knockdown CK1δ in two human EOC cell lines, OVCAR3 and IGROV1 cells. To this end, we transduced cells with lentiviral vectors bearing shRNA directed against *CSNK1D*, named sh599 and sh1552; control cells were generated by transduction with scramble shRNA (shCTRL). *CSNK1D* specific shRNA significantly downregulated CK1δ at both mRNA (Supplementary Figures 1A,B) and protein levels (Supplementary Figures 1C,D) in both cell lines.

**Figure 2:**
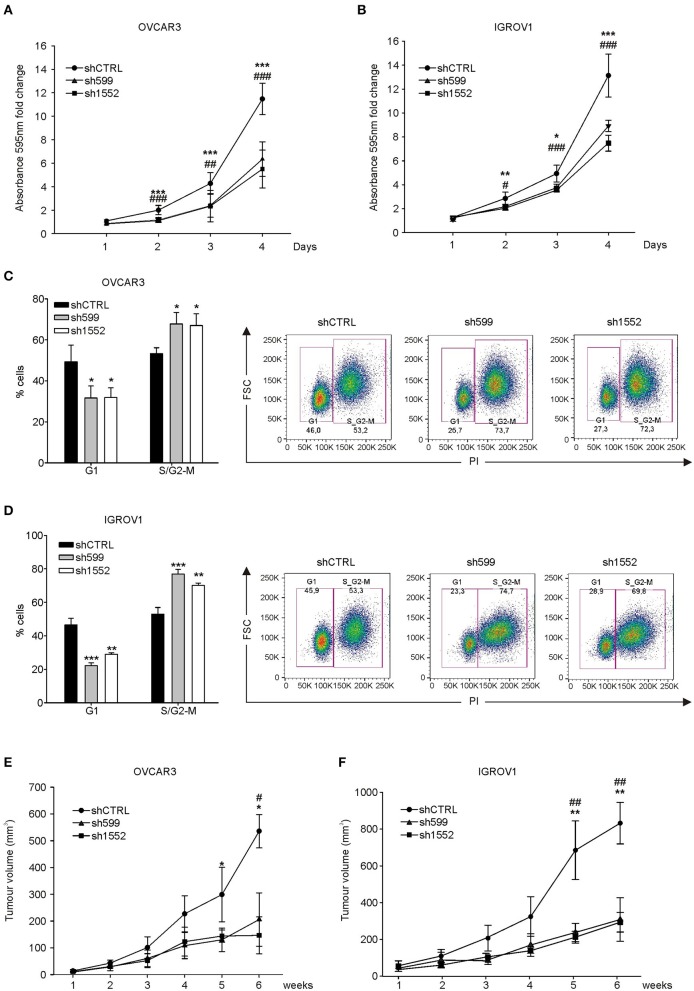
CK1δ knockdown affects human ovarian cancer cell proliferation. **(A,B)** Growth curves of shCTRL, sh599, and sh1552 OVCAR3 **(A)** and IGROV1 **(B)** cells determined by crystal violet proliferation assay. Crystal violet absorbance (595 nm) was normalized to T1. Data are expressed as the mean ± S.D. (N=6). *, ^#^*p* < 0.05; **, ^*##*^*p* < 0.001; ***, ^*###*^*p* < 0.001; *sh599 vs. shCTRL; ^#^sh1552 vs. shCTRL. **(C,D)** Cell cycle analysis of EdU-positive shCTRL, sh599, and sh1552 OVCAR3 **(C)** and IGROV1 **(D)** cells performed after 8 and 7 h culture, respectively. On the left, graphs represent the mean ± S.D (*N* = 3). *p < 0.05; **p < 0.01; ***p < 0.001. On the right, representative dot plots are shown. **(E,F)** Growth curves of tumors generated by shCTRL, sh599, and sh1552 OVCAR3 **(E)** and IGROV1 **(F)** cells after s.c. injection in NSG mice. Data are expressed as the mean ± S.D (*N* = 5 mice/experimental group). *, ^#^*p* < 0.05; **, ^*##*^*p* < 0.001; *sh599 vs. shCTRL; ^#^sh1552 vs. shCTRL.

Afterwards, cell growth was assessed by proliferation assay. Growth curves showed that CK1δ knockdown significantly affected both cell lines in their proliferative potential (Figures 2A,B). However, the observed differences in cell proliferation rate were not due to alterations in cell viability linked to CK1δ knockdown, since no significant differences were detected in the apoptotic cell rate (Annexin V-positive cells, Supplementary Figure 2). Furthermore, EdU-labeled cells were let proceed through the cell cycle for a few hours (7–8 h) and then analyzed with propidium iodide staining to detect their distribution in the different phases. According to the proliferation assay, cell cycle analysis highlighted that CK1δ knocked-down OVCAR3 (Figure 2C) and IGROV1 (Figure 2D) cells progressed more slowly through cell cycle phases than control cells. Indeed, after EdU incorporation, CK1δ knocked-down OVCAR3 and IGROV1 cells displayed a lower percentage of cells in G1 and a higher percentage in S/G2-M compared to shCTRL, meaning a braking of the progression through the cell cycle.

Eventually, we assessed the *in vivo* tumor growth potential of knocked-down OVCAR3 and IGROV1 cells injected s.c. into NSG mice. All cell lines were able to generate tumors, but in agreement with *in vitro* experiments, and with previously published data (30), sh599 and sh1552 tumors grew slower compared to shCTRL ones (Figures 2E,F). CK1δ silencing efficiency was checked at the end of the experiment, confirming that CK1δ was still knocked-down (Supplementary Figure 3).

### CK1δ Knockdown Is Associated With Sensitization to Carboplatin Treatment Mediated by p21 and XIAP Downregulation

**Figure 3:**
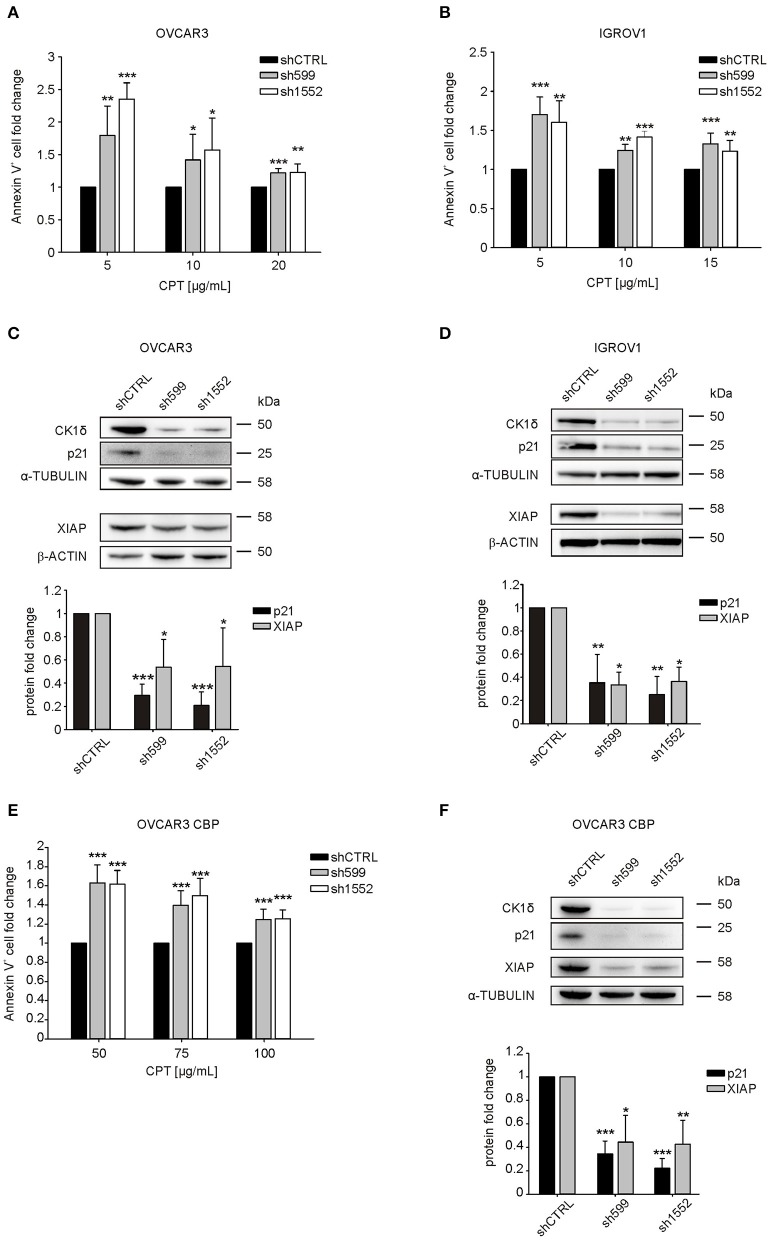
CK1δ knockdown sensitizes human ovarian cancer cells to CPT treatment. **(A, B, E)** ShCTRL, sh599, and sh1552 OVCAR3 **(A)**, IGROV1 **(B)**, and OVCAR3 CBP **(E)** cells were challenged with scalar doses of CPT for 72 h. Apoptosis was then assayed by Annexin-V staining. The graphs represent the mean ± S.D (*N* = 3). Data were normalized to the corresponding shCTRL. *p < 0.05; **p < 0.01; ***p < 0.001 **(C,D,F)** WB analysis of p21 andXIAP in shCTRL, sh599, and sh1552 OVCAR3 **(C)**, IGROV1 **(D)**, and OVCAR3 CBP **(F)** cells. Signals were normalized to α-tubulin or β-actin, as indicated. On the top, representative blots. On the bottom, graphs represent the mean ± S.D (*N* = 3). *p < 0.05; **p < 0.01; ***p < 0.001.

Since CK1δ has a role in the regulation of DNA-damage response and apoptosis (14), we tested knocked-down and control cell sensitivity to carboplatin (CPT), an alkylating agent used as first line chemotherapy for ovarian cancer treatment (32). Interestingly, after 72 h CPT treatment, we observed a significant increase in the percentage of apoptotic cells (Annexin V-positive) in CK1δ knocked-down OVCAR3 and IGROV1 cell lines (Figures 3A,B).

P21(Cip1/Waf1) is known to protect against apoptosis induced by DNA damage following radiation and cytotoxic agents, and to be involved in DNA repair (33), hence we wondered whether its expression was affected in CK1δ knocked-down, CPT-sensitive OVCAR3 and IGROV1 cells. Notably, we observed a significant decrease in p21 protein expression following CK1δ knockdown in both cell lines (Figures 3C,D).

Finally, we sought to further characterize the molecular basis underlying CPT cell sensitization. Thus, we checked the expression of X-linked inhibitor of apoptosis protein (XIAP), a member of the family of anti-apoptotic proteins, whose overexpression in cancer cells is associated to resistance to a variety of apoptotic stimuli (34, 35).

Indeed, previous literature showed that the CK1 inhibition and CK2 silencing brought to XIAP downregulation, thus sensitizing cells to pro-apoptotic stimuli (19, 36, 37). Accordingly, we found that XIAP was significantly downregulated in CK1δ knocked-down cells (Figures 3C,D). This result is not in contrast with the absence of difference in cell viability between CK1δ-deficient and proficient cells in basal culture conditions (Supplementary Figure 2). Indeed, previous works already showed that XIAP downregulation does not induce basal cell death by itself, but only in the presence of pro-apoptotic stimuli that lead to caspase activation (38, 39).

In order to further prove the role of CK1δ in the response of ovarian cancer cells to CPT, we silenced CK1δ in CPT-resistant OVCAR3 cells (OVCAR3 CBP, IC_50_ 75 μg/mL), obtained from parental OVCAR3 cell line (IC_50_ 15 μg/mL) by 72 h exposure to increasing doses of CPT (21). Notably, in agreement with previous results shown in Figure 3A, CK1δ-knocked down CPT-resistant cells (Figure 3F) displayed a significant higher percentage of apoptotic cells after 72 h CPT treatment compared to shCTRL cells (Figure 3E). Again, both p21 and XIAP resulted to be significantly downregulated in CK1δ-knocked down OVCAR3 CBP cells (Figure 3F). Similar results were also obtained in another CPT-resistant ovarian cancer cell line, MES-OV CBP (IC_50_ 75 μg/mL), obtained from parental MES-OV cell line (IC_50_ 20 μg/mL) by 72 h exposure to increasing doses of CPT (Supplementary Figures 4A,B) (21).

Therefore, we may conclude that CK1δ knockdown is functionally associated with p21(Cip1/Waf1) and XIAP downregulation, and this could be a possible explanation for ovarian cancer cell sensitization to CPT treatment, probably due to an impaired DNA-damage response and apoptosis control.

### CK1δ Knockdown Affects Migration in Human Ovarian Cancer Cells

**Figure 4:**
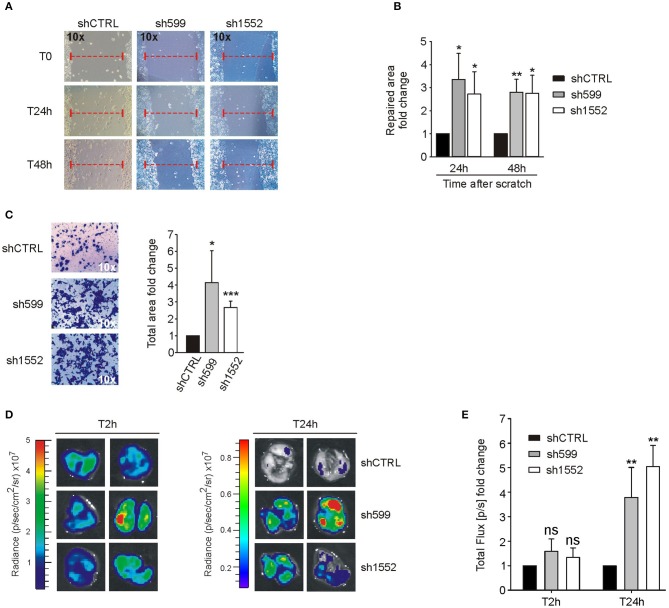
CK1δ knockdown enhances OVCAR3 cell motility. **(A,B)** Wound healing assay performed on shCTRL, sh599, and sh1552 OVCAR3 cells. Pictures of the scratch area were taken at T0 and after 24 and 48 h. Distance between the two sides of the scratch was quantified using ImageJ software. The repaired area was normalized to shCTRL. **(A)** One representative experiment is shown. **(B)** The graphs represent the mean of repaired area at T24 and T48h ± S.D (*N* = 3). *p < 0.05; **p < 0.01 **(C)** Transwell migration assay performed on shCTRL, sh599, and sh1552 OVCAR3 cells. On the left, representative pictures of migrated cells. On the right, the graphs represent the mean fold change of total area ± S.D (*N* = 3). *p < 0.05; ***p < 0.001 **(D,E)** Lung colonization assay. ShCTRL, sh599, and sh1552 Fluc-OVCAR3 were injected i.v. in NOD/SCID mice. Luciferin was administered to detect tumor cells. **(D)** *Ex vivo* imaging of lungs harvested at 2 (T2h) and 24 h (T24h) after i.v. injection. Representative pictures are shown. **(E)** The graph represents the mean of bioluminescence signals ± S.E.M (*N* = 5 mice/experimental group), normalized to shCTRL group. **p < 0.01; ns, not significant.

Since CK1δ has been shown to regulate cell migration of triple negative breast cancer cells (40), we verified its possible involvement in the modulation of ovarian cancer cell motility. For this purpose, we firstly performed wound healing assay. To exclude biases due to cell proliferation, cells were maintained in serum-free medium. Interestingly, sh599 and sh1552 OVCAR3 cells were able to cover a larger area than shCTRL cells after 24 and 48 h (Figures 4A,B). Therefore, CK1δ knocked-down OVCAR3 cells displayed a higher migratory capacity.

This finding was further confirmed by transwell migration assay. Indeed, sh599 and sh1552 OVCAR3 cells passed through the transwell filter at a higher extent than shCTRL cells (Figure 4C).

**Figure 5:**
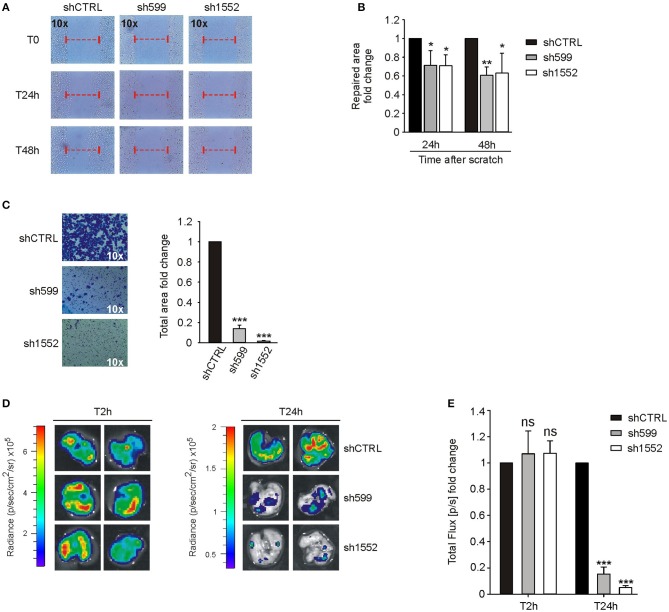
CK1δ knockdown impairs IGROV1 cell motility. **(A,B)** Wound healing assay performed on shCTRL, sh599, and sh1552 IGROV1 cells. Pictures of the scratch area were taken at T0 and after 24 and 48 h. Distance between the two sides of the scratch was quantified using ImageJ software. The repaired area was normalized to shCTRL. **(A)** One representative experiment is shown. **(B)** The graphs represent the mean of repaired area at T24 and T48h ± S.D (*N* = 3). *p < 0.05; **p < 0.01 **(C)** Transwell migration assay performed on shCTRL, sh599, and sh1552 IGROV1 cells. On the left, representative pictures of migrated cells. On the right, the graphs represent the mean fold change of total area ± S.D (*N* = 3). ***p < 0.001 **(D,E)** Lung colonization assay. ShCTRL, sh599, and sh1552 Fluc-IGROV1 were injected i.v. in NOD/SCID mice. Luciferin was administered to detect tumor cells. **(D)** *Ex vivo* imaging of lungs harvested at 2 (T2h) and 24 h (T24h) after i.v. injection. Representative pictures are shown. **(E)** The graph represents the mean of bioluminescence signals ± S.E.M. (*N* = 5 mice/experimental group), normalized to shCTRL group. ***p < 0.001; ns, not significant.

On the contrary, wound healing (Figures 5A,B) and transwell migration assays (Figure 5C) showed that sh599 and sh1552 IGROV1 cells migrated at a lower extent than shCTRL cells.

Moreover, since homing in secondary organs is a common trait of cells endowed with metastatic potential, we also performed an *in vivo* short-term experimental metastasis assay, in order to exclude any effect due to differences in cell proliferation, as already shown in Figures 2A–F. To this aim, we evaluated lung colonization ability of CK1δ knocked-down OVCAR3 and IGROV1 cells. First, shCTRL, sh599 and sh1552 cells were transduced with firefly luciferase in order to make cells detectable *in vivo*. Subsequently, cells were injected into the tail vein of immunocompromised NOD/SCID mice. Imaging performed after 2 h from injection showed no significant differences among cell lines, while 24 h after injection a higher bioluminescence signal was observed in the lungs harvested from mice injected with sh599 or sh1552 OVCAR3 cells (Figures 4D,E), further proving that CK1δ ablation increased the homing potential of OVCAR3 cells.

According to the above *in vitro* data, *in vivo* lung colonization assay confirmed that sh599 and sh1552 IGROV1 cells have an impaired homing capacity as compared to shCTRL cells (Figures 5D,E).

**Figure 6:**
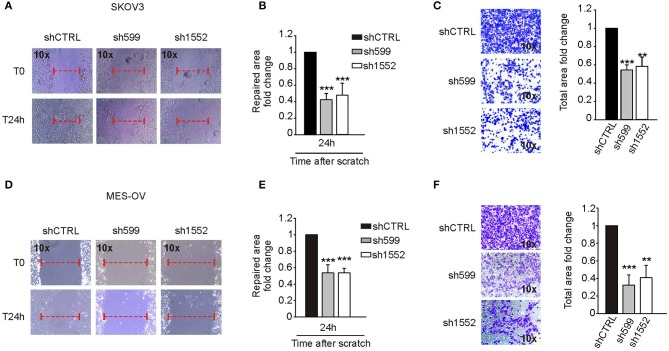
CK1δ knockdown impairs SKOV3 and MES-OV cell motility. **(A,B; D,E)** Wound healing assay performed on shCTRL, sh599, and sh1552 SKOV3 **(A,B)** and MES-OV **(D,E)** cells. Pictures of the scratch area were taken at T0 and after 24 h. Distance between the two sides of the scratch was quantified using ImageJ software. The repaired area was normalized to shCTRL. **(A,D)** One representative experiment is shown. **(B,E)** The graphs represent the mean of repaired area at T24 ± S.D. (*N* = 4). ***p < 0.001 **(C,F)** Transwell migration assay performed on shCTRL, sh599, and sh1552 SKOV3 **(C)** and MES-OV **(F)** cells. On the left, representative pictures of migrated cells. On the right, the graphs represent the mean fold change of total area ± S.D. (*N* = 3). **p < 0.01; ***p < 0.001.

In view of the contrasting results described above, we decided to investigate more deeply CK1δ role in cell motility. To this end, we knocked down CK1δ in two additional ovarian cancer cell lines, SKOV3 and MES-OV (Supplementary Figures 5A,B). In agreement with what observed for IGROV1 cells, both cell lines displayed an impaired migratory capability in both wound healing (Figures 6A,B,D,E) and transwell migration (Figures 6C,F) assays.

To sum up, the available data suggest that CK1δ-knockdown influences negatively ovarian cancer cell migratory and homing capability, but some exceptions could be present due to the complex cellular context.

## Discussion

EOC carcinogenesis is supported by numerous genetic and epigenetic alterations and aberrant signaling molecule activation (41–47). Due to its reported genetic amplification in EOC and its tumor-favoring role described in different cancers (8), we focused on CK1δ, a member of a kinase family characterized by pleiotropic cellular functions, including cell cycle progression, p53 control, mitotic spindle arrangement, and circadian rhythm protein turnover (6). Interestingly, proteins involved in circadian rhythm control, e.g., PER, CRY, BMAL1, CLOCK, are highly expressed in the ovaries, where they regulate ovulation and hormonal cycles in general (43, 48), and alterations in their expression levels are associated with an increased risk of ovarian cancer and affect cancer growth, invasiveness, and drug sensitivity (49–51).

Previous work highlighted a negative impact of CK1δ inhibition on cell cycle progression and proper mitosis, eventually leading to apoptosis (18, 20, 29, 52). Moreover, *in vivo* breast and pancreatic tumor growth was delayed by CK1δ inhibitor administration (19, 30), and recently, impaired cell migration and metastases of triple negative breast cancer were also assessed (40).

Since CK1δ resulted to be expressed at a higher extent in ovarian cancer tissue compared to the normal counterpart at both mRNA (Oncomine™) and protein levels (The Human Protein Atlas), we investigated its potential role in the regulation of EOC cellular functions.

In agreement with Oncomine™ and The Human Protein Atlas data, EOC cell lines, PDOVCA, and PDX samples displayed higher CK1δ levels in all the specimens compared to the non-tumoral counterpart. Genetic ablation of CK1δ in OVCAR3 and IGROV1 ovarian cancer cells impaired cell proliferation in both cell lines, and accordingly, CK1δ-deficient cells gave rise to smaller tumors in immunocompromised mice in agreement with previous literature (19, 30).

P21(Cip1/Waf1) is a well-known tumor-suppressor that arrests cell cycle progression by disrupting CDK/cyclins complexes and by associating to proliferating cell nuclear antigen (PCNA) (53). Nonetheless, high p21 protein levels have been shown to convey mitogenic signals in vascular smooth muscle cells, augmenting the transit through cell cycle (54).

Besides its role in cell cycle control, p21 (Cip1/Waf1) is involved in DNA repair (33) and it determines an apoptosis-resistant phenotype, acting as a tumor-promoting factor (55). For instance, renal cancer is usually difficult to treat because chemo-resistant, due to a highly effective DNA repair response mediated by high p21 levels (56). P21 attenuation by antisense oligonucleotides (57), small molecules (56), or sorafenib (58), is associated to increased chemo-sensitivity, with higher apoptosis rates after doxorubicin, paclitaxel and cisplatin treatment. In prostate cancer, infiltrating mast cells induced p21 expression, thus increasing docetaxel resistance (59). Furthermore, Pavan et al. (60) reported that IRF-1 knockdown sensitized ovarian cancer cells to cisplatin because of impaired p21 induction. Accordingly, CK1δ knocked-down CPT-sensitive and resistant cells showed p21 attenuation and were similarly sensitized to CPT treatment, displaying a higher proportion of apoptotic cells than control ones.

XIAP is a member of the “inhibitor of apoptosis” family, a group of proteins characterized by the presence of baculoviral IAP repeats, responsible for their inhibitory binding to caspases. XIAP in particular can bind both initiator and effector caspases (61).

Izeradjene et al. described XIAP downregulation in colorectal cancer cells following the administration of the CK1 inhibitor CKI-7 only if combined with TRAIL (37) and in CK2α knocked-down rhabdomyosarcoma cells (36). Furthermore, Brockschmidt et al. (19) reported that CK1δ/ε inhibitor IC261 exerted pro-apoptotic effects in pancreatic ductal adenocarcinoma by reducing the expression of many anti-apoptotic proteins, including XIAP. Moreover, even though McManus et al. (38) demonstrated that XIAP knockdown sensitized breast cancer cells to taxanes, but not to CPT, recent data by Zhang et al. (62) highlighted that XIAP high-expressing ovarian cancer patients were less likely to respond to CPT treatment and showed that XIAP knockdown in ovarian cancer cell lines slowed their proliferation kinetics, on one hand, and sensitized them to this drug *in vitro*, on the other. Previously, Asselin et al. (63) demonstrated that XIAP overexpression prevented cisplatin-induced apoptosis in A2780 ovarian cancer cell line in a PI3K/Akt-dependent manner. Accordingly, we observed a reduction in XIAP expression in CK1δ knocked-down CPT-sensitive and resistant cells, sensitized to carboplatin treatment.

A recent paper reported that CK1δ knockdown impaired breast cancer cell migration and invasion and upregulated epithelial markers such as claudin1 and occludin (40). Accordingly, specific knockdown of the CK1δ highly related kinase, CK1ε, demonstrated a 60–80% reduction in the migration ability of the EOC cell line SKOV3 (64).

Likewise, different migration assays performed on IGROV1, SKOV3, and MES-OV cells revealed a reduced migratory capacity of CK1δ knocked-down cells. On the contrary, the opposite outcome was observed when the same experiments were performed on OVCAR3 cells. Indeed, sh599 and sh1552 OVCAR3 cells presented a higher motility both *in vitro* and *in vivo*. On the whole, our experiments, and previous literature, support a positive role of CK1δ in cancer cell migration. However, an opposite behavior could also be observed. Likely, this kinase interacts directly or indirectly with different partners depending on the complex cellular context, and further work is required to unveil the molecular mechanism underlying this CK1δ-dependent phenomenon.

In summary, we have identified CK1δ as an important player in the regulation of cell proliferation, response to chemotherapeutic drugs, and migration in ovarian cancer cells. Our results would suggest CK1δ as an attractive target for ovarian cancer treatment, but further investigation regarding its role in migration is recommended before the introduction of CK1δ inhibitors into the clinics for EOC management.

## Data Availability Statement

The expression data of CK1δ gene (*CSNK1D*) and protein were obtained from Oncomine™ and The Human Protein Atlas platforms, respectively. Data and images can be found at the following links: https://www.oncomine.org, https://www.proteinatlas.org/ENSG00000141551-CSNK1D/tissue/ovary#img, and https://www.proteinatlas.org/ENSG00000141551-CSNK1D/pathology/tissue/ovarian$+$cancer#img.

The raw data supporting the conclusions of this manuscript will be made available by the authors, without undue reservation, to any qualified researcher.

## Ethics Statement

The studies involving human participants were reviewed and approved by IOV Institutional Review Board and Ethics Committee. The patients/participants provided their written informed consent to participate in this study. The animal study was reviewed and approved by Italian Ministry of Health (authorization n° 250/2017-PR).

## Author Contributions

AA, SP, and EM designed the experiments. SP, EM, AP, EC, and GP performed the experiments and analyzed the results. VB helped perform experiments. SP and EM wrote the manuscript. All authors read and approved the manuscript for publication.

### Conflict of Interest

The authors declare that the research was conducted in the absence of any commercial or financial relationships that could be construed as a potential conflict of interest.

## Footnotes

^1^https://www.oncomine.org

^2^https://www.proteinatlas.org

^3^https://www.proteinatlas.org/ENSG00000141551-CSNK1D/tissue/ovary#img

^4^https://www.proteinatlas.org/ENSG00000141551-CSNK1D/pathology/tissue/ovarian+cancer#img

^5^https://www.oncomine.org

^6^https://www.proteinatlas.org

## References

[1] Siegel RL Miller KD Jemal A Cancer statistics, 2018. CA Cancer J Clin. (2018) 68:7–30. 10.3322/caac.21442 29313949

[2] Jacobs IJ Menon U. Progress and challenges in screening for early detection of ovarian cancer. Mol Cell Proteomics. (2004) 3:355–66. 10.1074/mcp.R400006-MCP200 14764655

[3] Lim HJ Ledger W Targeted therapy in ovarian cancer. Womens Health. (2016) 12:363–78. 10.2217/whe.16.4 PMC5384518 27215391

[4] Chen Y Du H. The promising PARP inhibitors in ovarian cancer therapy: from olaparib to others. Biomed Pharmacother. (2018) 99:552–60. 10.1016/j.biopha.2018.01.094 29895102

[5] Francesco P Sabrina M Anna C Laura Quotti T Gurrieri C Gianpietro S Serine-threonine protein kinases CK1, CK2 and GSK3 in normal and malignant haematopoiesis. Curr Signal Trans Ther. (2011) 6:88–98. 10.2174/157436211794109370

[6] Knippschild U Gocht A Wolff S Huber N Lohler J Stoter M. The casein kinase 1 family: participation in multiple cellular processes in eukaryotes. Cell Signal. (2005) 17:675–89. 10.1016/j.cellsig.2004.12.011 15722192

[7] Bischof J Randoll SJ Sussner N Henne-Bruns D Pinna LA Knippschild U. CK1delta kinase activity is modulated by Chk1-mediated phosphorylation. PLoS ONE. (2013) 8:e68803. 10.1371/journal.pone.0068803 23861943 PMC3701638

[8] Schittek B Sinnberg T. Biological functions of casein kinase 1 isoforms and putative roles in tumorigenesis. Mol Cancer. (2014) 13:231. 10.1186/1476-4598-13-231 25306547 PMC4201705

[9] Zhang L Jia J Wang B Amanai K Wharton KA Jr Jiang J. Regulation of wingless signaling by the CKI family in *Drosophila* limb development. Dev Biol. (2006) 299:221–37. 10.1016/j.ydbio.2006.07.025 16987508 PMC1855185

[10] Jiang J. CK1 in developmental signaling: Hedgehog and Wnt. Curr Top Dev Biol. (2017) 123:303–29. 10.1016/bs.ctdb.2016.09.002 28236970 PMC5837819

[11] Penas C Ramachandran V Simanski S Lee C Madoux F Rahaim RJ. Casein kinase 1delta-dependent Wee1 protein degradation. J Biol Chem. (2014) 289:18893–903. 10.1074/jbc.M114.547661 24817118 PMC4081930

[12] Greer YE Gao B Yang Y Nussenzweig A Rubin JS. Lack of casein kinase 1 delta promotes genomic instability–the accumulation of DNA damage and down-regulation of checkpoint kinase 1. PLoS ONE. (2017) 12:e0170903. 10.1371/journal.pone.0170903 28125685 PMC5268481

[13] Knippschild U Wolff S Giamas G Brockschmidt C Wittau M Wurl PU. The role of the casein kinase 1 (CK1) family in different signaling pathways linked to cancer development. Onkologie. (2005) 28:508–14. 10.1159/000087137 16186692

[14] Knippschild U Kruger M Richter J Xu P Garcia-Reyes B Peifer C. The CK1 family: contribution to cellular stress response and its role in carcinogenesis. Front Oncol. (2014) 4:96. 10.3389/fonc.2014.00096 24904820 PMC4032983

[15] Cerami E Gao J Dogrusoz U Gross BE Sumer SO Aksoy BA. The cBio cancer genomics portal: an open platform for exploring multidimensional cancer genomics data. Cancer Discov. (2012) 2:401–4. 10.1158/2159-8290.CD-12-0095 22588877 PMC3956037

[16] Gao J Aksoy BA Dogrusoz U Dresdner G Gross B Sumer SO. Integrative analysis of complex cancer genomics and clinical profiles using the cBioPortal. Sci Signal. (2013) 6:pl1. 10.1126/scisignal.2004088 23550210 PMC4160307

[17] Maritzen T Lohler J Deppert W Knippschild U. Casein kinase I delta (CKIdelta) is involved in lymphocyte physiology. Eur J Cell Biol. (2003) 82:369–78. 10.1078/0171-9335-00323 12924632

[18] Stoter M Bamberger AM Aslan B Kurth M Speidel D Loning T. Inhibition of casein kinase I delta alters mitotic spindle formation and induces apoptosis in trophoblast cells. Oncogene. (2005) 24:7964–75. 10.1038/sj.onc.1208941 16027726

[19] Brockschmidt C Hirner H Huber N Eismann T Hillenbrand A Giamas G. Anti-apoptotic and growth-stimulatory functions of CK1 delta and epsilon in ductal adenocarcinoma of the pancreas are inhibited by IC261 *in vitro* and *in vivo*. Gut. (2008) 57:799–806. 10.1136/gut.2007.123695 18203806

[20] Bibian M Rahaim RJ Choi JY Noguchi Y Schurer S Chen W. Development of highly selective casein kinase 1delta/1epsilon (CK1delta/epsilon) inhibitors with potent antiproliferative properties. Bioorg Med Chem Lett. (2013) 23:4374–80. 10.1016/j.bmcl.2013.05.075 23787102 PMC3783656

[21] Brozovic A. The relationship between platinum drug resistance and epithelial-mesenchymal transition. Arch Toxicol. (2017) 91:605–19. 10.1007/s00204-016-1912-7 28032148

[22] Rhodes DR Yu J Shanker K Deshpande N Varambally R Ghosh D. ONCOMINE: a cancer microarray database and integrated data-mining platform. Neoplasia. (2004) 6:1–6. 10.1016/S1476-5586(04)80047-2 15068665 PMC1635162

[23] Uhlen M Fagerberg L Hallstrom BM Lindskog C Oksvold P Mardinoglu A. Proteomics. Tissue-Based Map of the human proteome. Science. (2015) 347:1260419. 10.1126/science.1260419 25613900

[24] Mazzoldi EL Pavan S Pilotto G Leone K Pagotto A Frezzini S. A juxtacrine/paracrine loop between C-Kit and stem cell factor promotes cancer stem cell survival in epithelial ovarian cancer. Cell Death Dis. (2019) 10:412. 10.1038/s41419-019-1656-4 31138788 PMC6538673

[25] Pavan S Musiani D Torchiaro E Migliardi G Gai M Di Cunto F. HSP27 is required for invasion and metastasis triggered by hepatocyte growth factor. Int J Cancer. (2014) 134:1289–99. 10.1002/ijc.28464 23996744

[26] Pagotto A Pilotto G Mazzoldi EL Nicoletto MO Frezzini S Pasto A. Autophagy inhibition reduces chemoresistance and tumorigenic potential of human ovarian cancer stem cells. Cell Death Dis. (2017) 8:e2943. 10.1038/cddis.2017.327 28726781 PMC5550872

[27] Di Noto G Chiarini M Paolini L Mazzoldi EL Giustini V Radeghieri A. Immunoglobulin free light chains and GAGs mediate multiple myeloma extracellular vesicles uptake and secondary nfkappab nuclear translocation. Front Immunol. (2014) 5:517. 10.3389/fimmu.2014.00517 25386176 PMC4209816

[28] Justus CR Leffler N Ruiz-Echevarria M Yang LV *In vitro* cell migration and invasion assays. J Vis Exp. (2014) e51046 10.3791/51046 PMC4186330 24962652

[29] Richter J Ullah K Xu P Alscher V Blatz A Peifer C. Effects of altered expression and activity levels of CK1delta and varepsilon on tumor growth and survival of colorectal cancer patients. Int J Cancer. (2015) 136:2799–810. 10.1002/ijc.29346 25404202

[30] Rosenberg LH Lafitte M Quereda V Grant W Chen W Bibian M. Therapeutic targeting of casein kinase 1delta in breast cancer. Sci Transl Med. (2015) 7:318ra202. 10.1126/scitranslmed.aac8773 26676609 PMC4809734

[31] Merritt MA Bentink S Schwede M Iwanicki MP Quackenbush J Woo T. Gene expression signature of normal cell-of-origin predicts ovarian tumor outcomes. PLoS ONE. (2013) 8:e80314. 10.1371/journal.pone.0080314 24303006 PMC3841174

[32] Marchetti C Pisano C Facchini G Bruni GS Magazzino FP Losito S. First-line treatment of advanced ovarian cancer: current research and perspectives. Expert Rev Anticancer Ther. (2010) 10:47–60. 10.1586/era.09.167 20014885

[33] Georgakilas AG Martin OA Bonner WM. p21: a two-faced genome guardian. Trends Mol Med. (2017) 23:310–9. 10.1016/j.molmed.2017.02.001 28279624

[34] Notarbartolo M Cervello M Poma P Dusonchet L Meli M D'Alessandro N. Expression of the IAPs in multidrug resistant tumor cells. Oncol Rep. (2004) 11:133–6. 10.3892/or.11.1.133 14654915

[35] Tong Q-S Zheng L-D Wang L Zeng F-Q Chen F-M Dong J-H. Downregulation of XIAP expression induces apoptosis and enhances chemotherapeutic sensitivity in human gastric cancer cells. Cancer Gene Therapy. (2005) 12:509. 10.1038/sj.cgt.7700813 15706355

[36] Izeradjene K Douglas L Delaney A Houghton JA. Influence of casein kinase II in tumor necrosis factor-related apoptosis-inducing ligand-induced apoptosis in human rhabdomyosarcoma cells. Clin Cancer Res. (2004) 10:6650–60. 10.1158/1078-0432.CCR-04-0576 15475455

[37] Izeradjene K Douglas L Delaney AB Houghton JA. Casein kinase I attenuates tumor necrosis factor-related apoptosis-inducing ligand-induced apoptosis by regulating the recruitment of fas-associated death domain and procaspase-8 to the death-inducing signaling complex. Cancer Res. (2004) 64:8036–44. 10.1158/0008-5472.CAN-04-0762 15520213

[38] McManus DC Lefebvre CA Cherton-Horvat G St-Jean M Kandimalla ER Agrawal S. Loss of XIAP protein expression by RNAi and antisense approaches sensitizes cancer cells to functionally diverse chemotherapeutics. Oncogene. (2004) 23:8105–17. 10.1038/sj.onc.1207967 15378029

[39] Cai J Wang D Bai ZG Yin J Zhang J Zhang ZT The long non-coding RNA XIAP-AS1 promotes XIAP transcription by XIAP-AS1 interacting with Sp1 in gastric cancer cells. PLoS ONE. (2017) 12:e0182433 10.1371/journal.pone.0182433 28792527 PMC5549724

[40] Bar I Merhi A Larbanoix L Constant M Haussy S Laurent S. Silencing of casein kinase 1 delta reduces migration and metastasis of triple negative breast cancer cells. Oncotarget. (2018) 9:30821–36. 10.18632/oncotarget.25738 30112110 PMC6089398

[41] Gov E Kori M Arga KY. RNA-based ovarian cancer research from 'a gene to systems biomedicine' perspective. Syst Biol Reprod Med. (2017) 63:219–38. 10.1080/19396368.2017.1330368 28574782

[42] Xing BL Li T Tang ZH Jiao L Ge SM Qiang X. Cumulative methylation alternations of gene promoters and protein markers for diagnosis of epithelial ovarian cancer. Genet Mol Res. (2015) 14:4532–40. 10.4238/2015.May.4.11 25966226

[43] Jim HS Lin HY Tyrer JP Lawrenson K Dennis J Chornokur G. Common genetic variation in circadian rhythm genes and risk of epithelial ovarian cancer (EOC). J Genet Genome Res. (2015) 2:1–23. 10.23937/2378-3648/1410017 26807442 PMC4722961

[44] Caiola E Broggini M Marabese M. Genetic markers for prediction of treatment outcomes in ovarian cancer. Pharmacogenomics J. (2014) 14:401–10. 10.1038/tpj.2014.32 25001881

[45] Al Bakir M Gabra H. The molecular genetics of hereditary and sporadic ovarian cancer: implications for the future. Br Med Bull. (2014) 112:57–69. 10.1093/bmb/ldu034 25473022

[46] Kurman RJ Shih Ie M. Molecular pathogenesis and extraovarian origin of epithelial ovarian cancer–shifting the paradigm. Hum Pathol. (2011) 42:918–31. 10.1016/j.humpath.2011.03.003 21683865 PMC3148026

[47] Smolle E Taucher V Pichler M Petru E Lax S Haybaeck J. Targeting signaling pathways in epithelial ovarian cancer. Int J Mol Sci. (2013) 14:9536–55. 10.3390/ijms14059536 23644885 PMC3676798

[48] Fahrenkrug J Georg B Hannibal J Hindersson P Gras S. Diurnal rhythmicity of the clock genes Per1 and Per2 in the rat ovary. Endocrinology. (2006) 147:3769–76. 10.1210/en.2006-0305 16675517

[49] Sun Y Jin L Sui YX Han LL Liu JH. Circadian gene CLOCK affects drug-resistant gene expression and cell proliferation in ovarian cancer SKOV3/DDP cell lines through autophagy. Cancer Biother Radiopharm. (2017) 32:139–46. 10.1089/cbr.2016.2153 28514207

[50] Xu H Wang Z Mo G Chen H. Association between circadian gene CLOCK and cisplatin resistance in ovarian cancer cells: a preliminary study. Oncol Lett. (2018) 15:8945–50. 10.3892/ol.2018.8488 29844814 PMC5958788

[51] Wang Z Li L Wang Y. Effects of Per2 overexpression on growth inhibition and metastasis, and on MTA1, nm23-H1 and the autophagy-associated PI3K/PKB signaling pathway in nude mice xenograft models of ovarian cancer. Mol Med Rep. (2016) 13:4561–8. 10.3892/mmr.2016.5116 27082164 PMC4878548

[52] Behrend L Milne DM Stoter M Deppert W Campbell LE Meek DW. IC261, a specific inhibitor of the protein kinases casein kinase 1-delta and -epsilon, triggers the mitotic checkpoint and induces p53-dependent postmitotic effects. Oncogene. (2000) 19:5303–13. 10.1038/sj.onc.1203939 11103931

[53] Karimian A Ahmadi Y Yousefi B. Multiple functions of p21 in cell cycle, apoptosis and transcriptional regulation after DNA damage. DNA Repair. (2016) 42:63–71. 10.1016/j.dnarep.2016.04.008 27156098

[54] Dong Y Chi SL Borowsky AD Fan Y Weiss RH. Cytosolic p21Waf1/Cip1 increases cell cycle transit in vascular smooth muscle cells. Cell Signal. (2004) 16:263–9. 10.1016/S0898-6568(03)00136-0 14636896

[55] Asada M Yamada T Ichijo H Delia D Miyazono K Fukumuro K. Apoptosis inhibitory activity of cytoplasmic p21(Cip1/WAF1) in monocytic differentiation. Embo J. (1999) 18:1223–34. 10.1093/emboj/18.5.1223 10064589 PMC1171213

[56] Park SH Wang X Liu R Lam KS Weiss RH. High throughput screening of a small molecule one-bead-one-compound combinatorial library to identify attenuators of p21 as chemotherapy sensitizers. Cancer Biol Ther. (2008) 7:2015–22. 10.4161/cbt.7.12.7069 18981726

[57] Park SH Park JY Weiss RH. Antisense attenuation of p21 sensitizes kidney cancer to apoptosis in response to conventional DNA damaging chemotherapy associated with enhancement of phospho-p53. J Urol. (2008) 180:352–60. 10.1016/j.juro.2008.02.038 18499163 PMC2705194

[58] Inoue H Hwang SH Wecksler AT Hammock BD Weiss RH. Sorafenib attenuates p21 in kidney cancer cells and augments cell death in combination with DNA-damaging chemotherapy. Cancer Biol Ther. (2011) 12:827–36. 10.4161/cbt.12.9.17680 21878748 PMC3225758

[59] Xie H Li C Dang Q Chang LS Li L. Infiltrating mast cells increase prostate cancer chemotherapy and radiotherapy resistances via modulation of p38/p53/p21 and ATM signals. Oncotarget. (2016) 7:1341–53. 10.18632/oncotarget.6372 26625310 PMC4811464

[60] Pavan S Olivero M Cora D Di Renzo MF. IRF-1 expression is induced by cisplatin in ovarian cancer cells and limits drug effectiveness. Eur J Cancer. (2013) 49:964–73. 10.1016/j.ejca.2012.09.024 23079474

[61] Cossu F Milani M Mastrangelo E Lecis D. Targeting the BIR domains of inhibitor of apoptosis (IAP) proteins in cancer treatment. Comput Struct Biotechnol J. (2019) 17:142–50. 10.1016/j.csbj.2019.01.009 30766663 PMC6360406

[62] Zhang Y Huang F Luo Q Wu X Liu Z Chen H. Inhibition of XIAP increases carboplatin sensitivity in ovarian cancer. Onco Targets Ther. (2018) 11:8751–9. 10.2147/OTT.S171053 30584333 PMC6287417

[63] Asselin E Mills GB Tsang BK. XIAP regulates Akt activity and caspase-3-dependent cleavage during cisplatin-induced apoptosis in human ovarian epithelial cancer cells. Cancer Res. (2001) 61:1862–8. 11280739

[64] Rodriguez N Yang J Hasselblatt K Liu S Zhou Y Rauh-Hain JA. Casein kinase I epsilon interacts with mitochondrial proteins for the growth and survival of human ovarian cancer cells. EMBO Mol Med. (2012) 4:952–63. 10.1002/emmm.201101094 22707389 PMC3491827

