# Supplementary material for: Casein Kinase 1 Delta Regulates Cell Proliferation, Response to Chemotherapy and Migration in Human Ovarian Cancer Cells
Source: Front Oncol. 2019 Nov 12;9:1211. doi: 10.3389/fonc.2019.01211 (PMC6874158; doi:10.3389/fonc.2019.01211)
Supplement: Supplementary file 1 [file Data_Sheet_1.DOCX]

***Supplementary material***

**2 Supplementary Figures and Tables**

**2.1 Supplementary Figures**

**
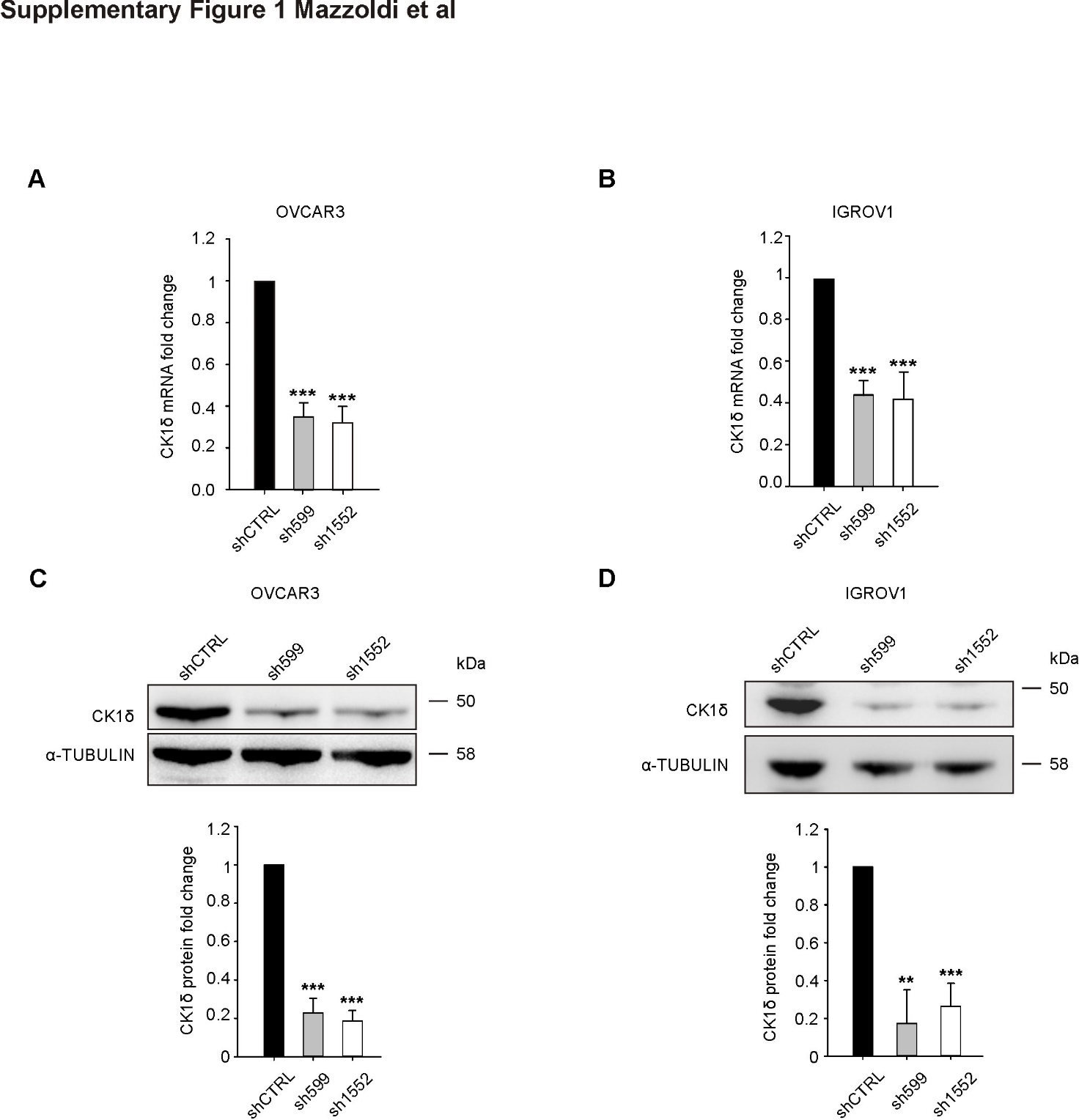
**

**Supplementary Figure 1. (A-B)** qRT-PCR analysis of CK1δ knockdown in OVCAR3 (**A**) and IGROV1 (**B**) cells. Data were normalized to shCTRL cells. Graphs represent the mean ± S.D. (N=5). *** p<0.001 **(C-D)** WB analysis of CK1δ knockdown in OVCAR3 (**C**) and IGROV1 (**D**) cells. Signals were normalized to α-tubulin. On the top, representative blots. On the bottom, graphs represent the mean ± S.D. (N=3). ** p<0.01; *** p<0.001

***
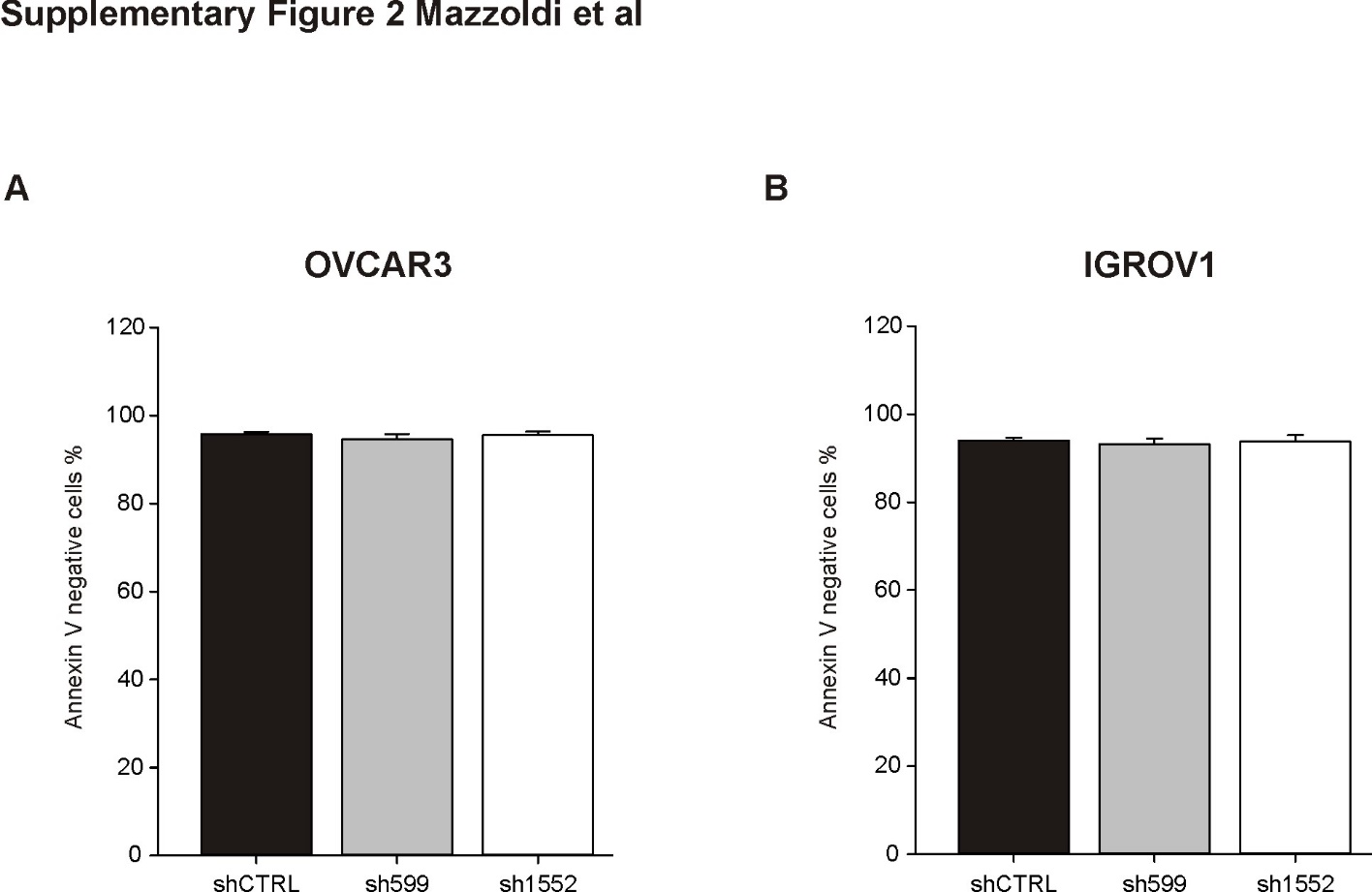
***

**Supplementary Figure 2.** OVCAR3 (**A**) and IGROV1 (**B**) cell viability after CK1δ knockdown. Apoptosis was assayed by Annexin-V staining. The graphs represent the mean ± S.D. (N=5).


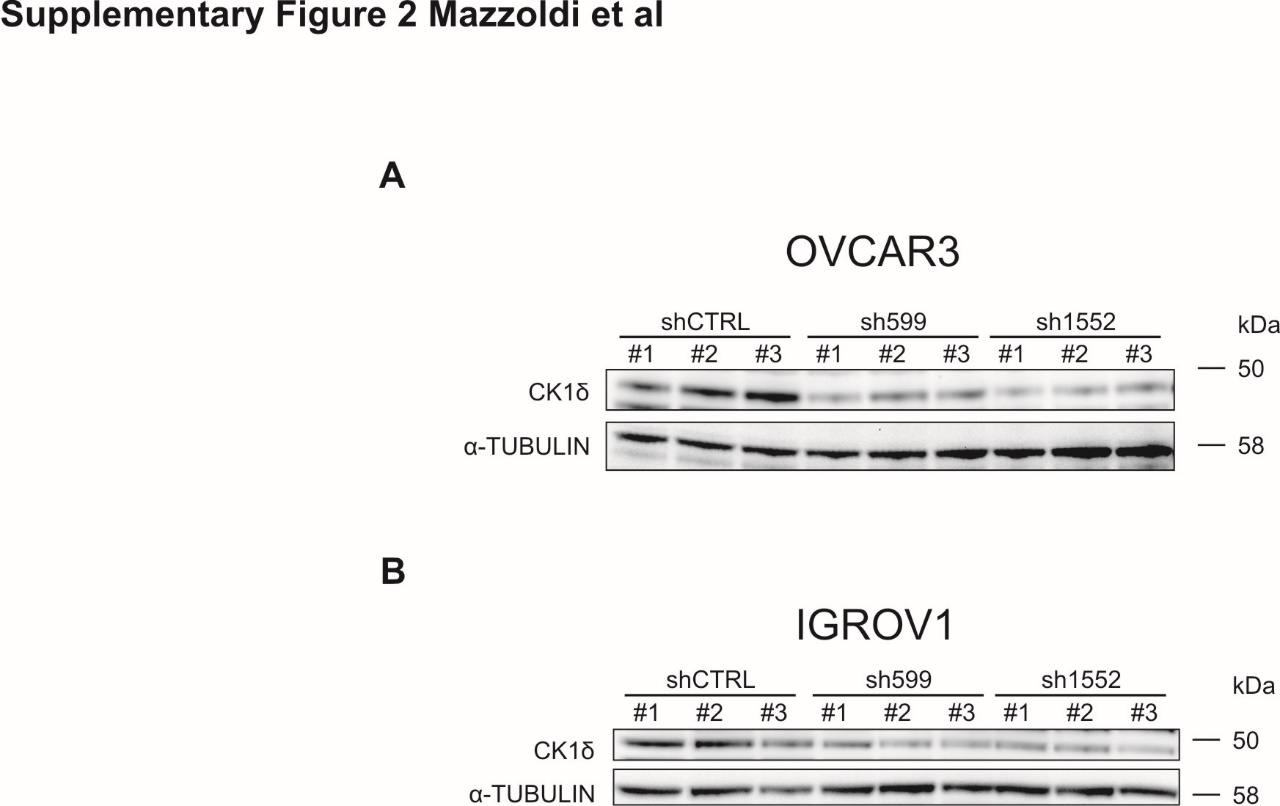


**Supplementary Figure 3.** WB analysis of CK1δ knockdown in OVCAR3 (**A**) and IGROV1 (**B**) tumors harvested at the end of the experiment reported in Fig. 2E and F.


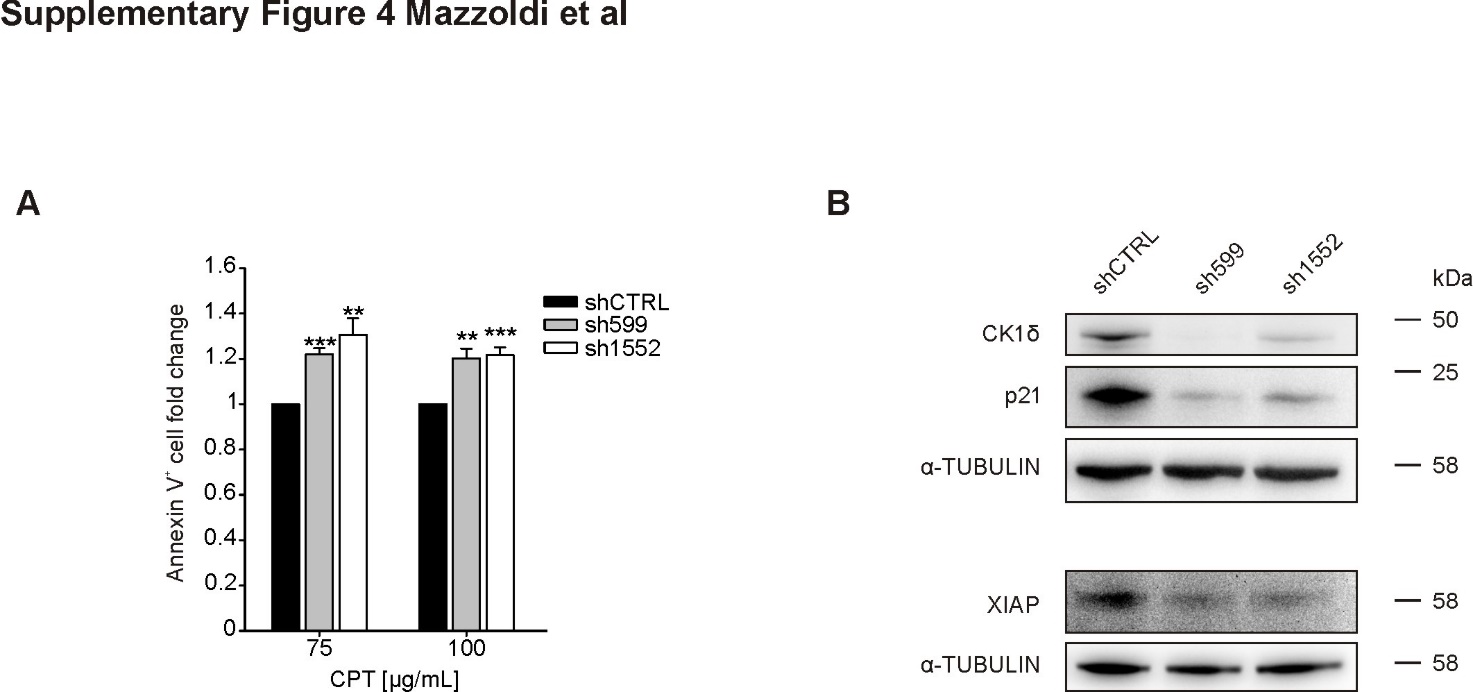


**Supplementary Figure 4. (A)** ShCTRL, sh599, and sh1552 MES-OV CBP cells were challenged with 75 or 100 µg/mL of CPT for 72h. Apoptosis was then assayed by Annexin-V staining. The graph represents the mean ± S.D. (N=3). Data were normalized to the corresponding shCTRL. **p<0.01; ***p<0.001 **(B)** WB analysis of CK1δ, p21, and XIAP in shCTRL, sh599, and sh1552 MES-OV CBP cells.


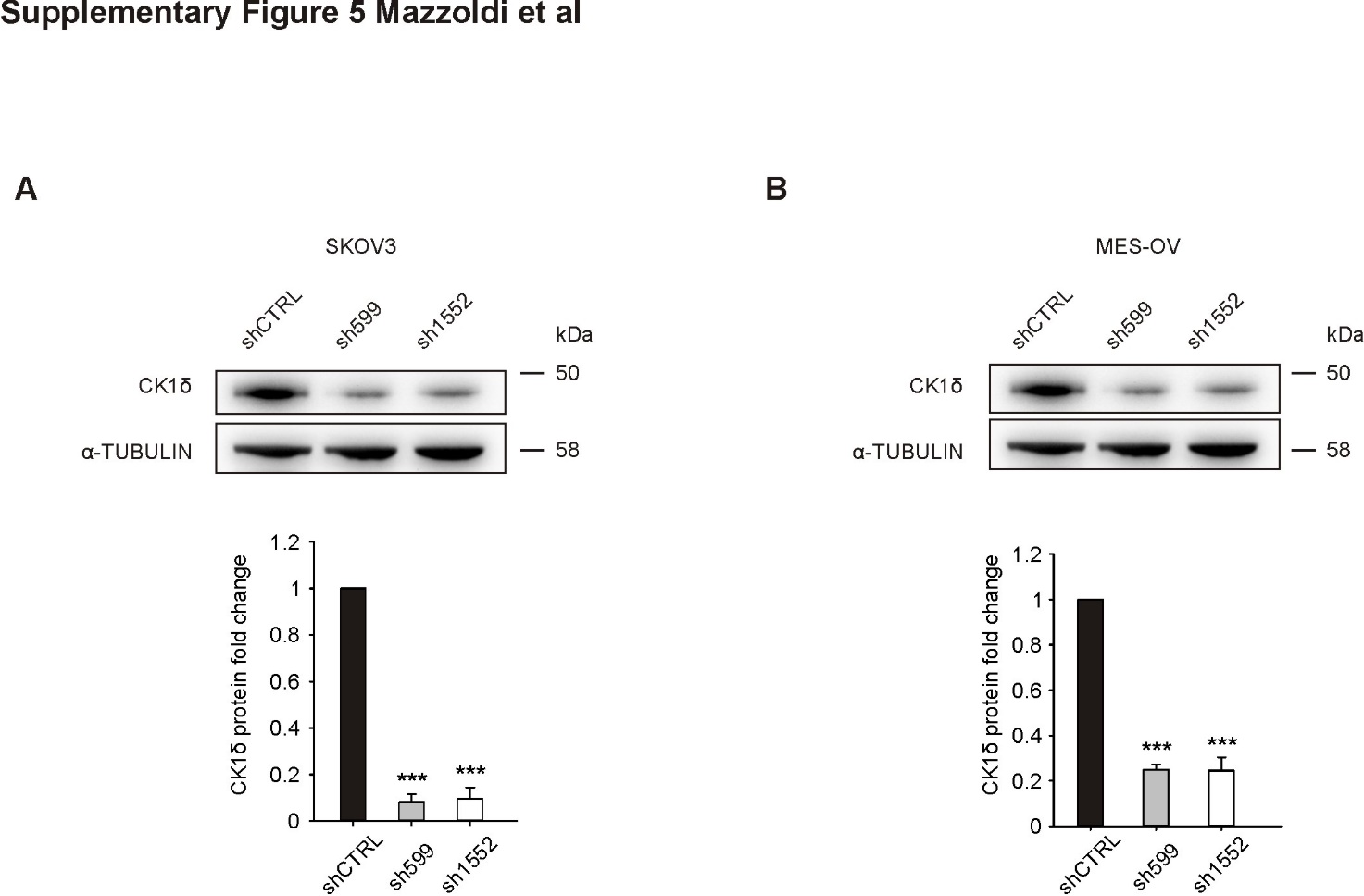


**Supplementary Figure 5.** WB analysis of CK1δ knockdown in SKOV3 (**A**) and MES-OV (**B**) cells. Signals were normalized to α-tubulin. On the top, representative blots. On the bottom, graphs represent the mean ± S.D. (N=3). *** p<0.001
